# Forty years of *The Selfish Gene* are not enough

**DOI:** 10.1186/s13059-016-0910-7

**Published:** 2016-03-02

**Authors:** Itai Yanai, Martin J. Lercher

**Affiliations:** Department of Biology, Technion - Israel Institute of Technion, Haifa, 32000 Israel; Institute for Computer Science, Heinrich Heine University, 40225 Düsseldorf, Germany

There is no book quite like Richard Dawkins’ *The Selfish Gene*. Forty years after its first publication, the book is still in Amazon’s top 10 for both the Genetics and Evolution categories, with over a million copies sold and more than 25 translated versions. Perhaps the best indicator of its enduring importance is its overwhelming influence on generations of scientists — including the authors of this piece — whom it inspired to explore genetics, genomics, and evolution. Where does the legacy of the book that took Darwin’s theory of evolution to its logical conclusion stand 40 years on?



Most importantly, Dawkins demonstrated with the utmost lucidity that we had biology upside down: evolution — and hence biology — is not concerned with the organism, but with the genes that survive unscathed through the eons by jumping from body to body. To bring this point home, he memorably defines bodies as survival machines: “robot vehicles blindly programmed to preserve the selfish molecules known as genes.” These survival machines — us — are discarded as our genes move on to another transient individual. And so, as Copernicus reoriented the solar system such that we are not at its center, and Darwin demoted humans to evolved great apes, Dawkins dealt the final blow to anthropocentrism: even we as individuals are not at the center of natural selection.

Dawkins wrote his book from the viewpoint of a zoologist, and he focused most of his attention on animal behavior. He demonstrates that many apparently paradoxical behaviors make perfect sense when looked at in the light of individual genes’ struggle to maximize their chances of perpetuation. For example, Dawkins recognized the muddle in human ethics over the level at which altruism is desirable — family, race, species, or all living things. From the viewpoint of the genes, the answer is easy: a gene benefits from favoring individuals who are likely to bear its copies. Popularizing groundbreaking work by his contemporaries, Dawkins explains the fine balance of competition and cooperation between siblings, between parents and their offspring, and between mates.

Although *The Selfish Gene* focuses on animals, over the past 20 years it has emerged that bacteria may provide more direct evidence for the book’s arguments. The first bacterial genome, that of *Haemophilus influenzae*, was sequenced in 1995 [1]; since then, thousands more have become available for analysis. Among the first orders of business in analyzing these genomes was the comparison of genes within and between species, allowing the delineation of a large number of gene families. Some genes were found to be universal to all genomes, such as the ribosome, polymerases, and certain DNA repair genes [2], giving testament to the “immortality” of genes.

But what is a gene, anyway? When we interviewed Dawkins last November, he described his pragmatic definition of a gene in the Mendelian sense: “it’s what persists through the generations.” Remarkably, when Dawkins wrote *The Selfish Gene*, no genome sequence had yet been fully deciphered; yet this did not stop him from deducing the genes’ workings at the genomic level. The most striking example may be Dawkins’ prediction of a phenomenon that turned out to be of truly genomic proportions: the existence of purely selfish DNA, genes that have no function other than to propagate themselves in the genome, for example, by copying and pasting themselves in a number of different chromosomes in our own genome and those of most walks of life. Twenty-five years after the book’s publication, the biggest discovery of the Human Genome Project was that half of our genome is indeed plastered with a plethora of such selfish elements, including a million short interspersed elements (SINEs), which are parasites of genomic parasites [3].

But gene families from bacteria revealed something even more far reaching. Before systematic genomic comparisons, we expected each gene to tell the history of its species. But the reality is far more complex. The vast majority of genes’ “profiles” — their presences or absences across strains and species — do not form tidy sub-branches on the tree of life, but are widely scattered. This is a telltale sign of rampant horizontal gene transfer. Genes that are involved in core metabolism or informational processing tend to stay put and their vertical inheritance is necessary for phylogenetic analysis of the species. By contrast, genes at the cell’s interface to the environment — for example, those involved in the metabolite import/export business or those working on the fringes of the great metabolic system — frequently jump from genome to genome [4].

There is often a rhythm and a rhyme to these gene migrations. A dramatic example is provided by the inhabitants of the genome of *Thermotoga maritima*, a bacterium that lives in extreme conditions such as hot springs and hydrothermal vents. A third of these genes have migrated into the *T. maritima* genome from archaeal genomes, where they had collaborated with their fellow genomic travelers to survive in the same extreme conditions [5]. Some genes travel in tight pairs, such as the restriction enzymes and their protective methyltransferases: when arriving in a new genome as a doublet, they bring with them the ability to destroy intruders while providing self-immunity from that same destruction. Gene migrations between genomes can occur exceedingly quickly: the gene sets of different *Escherichia coli* strains can differ much more than those of elephants and mice. In prokaryotes then, genes are easily distinguished by their evolutionary conservation, while genomes are rather more difficult to characterize. In our discussion with Dawkins, he noted that a version of *The Selfish Gene* focusing on bacterial genomics would be timely.

Despite its prominence, few important books have been misjudged by their title as much as *The Selfish Gene*. Even its readers tend to remember it by its title, which seems to imply that genes always behave as selfish entities. But that’s not even half of the story. *The Selfish Gene* delved deep into the issue of cooperation, both between individuals and between genes; as Dawkins himself writes on the first page, “my purpose is to examine the biology of selfishness and altruism.” Genes do not live in isolation, but depend on their environment to survive — that is, on the other genes in the genomes of their survival machines. To paraphrase Adam Smith’s observations on human societies, genes promote the wellbeing of their fellow genomic travelers and eventually of the whole gene pool, the “society of genes”, by observing their selfish interests [6].

How did such complex societies of genes evolve? As the genomes of more complex organisms such as *Caenorhabditis elegans*, *Drosophila*, and humans became available, a very different situation from that in bacteria emerged. In these organisms, the genome is very easy to define, but the gene is actually a blurry concept. For example, a gene in our genome might produce alternatively spliced variants, be regulated by very distant promoters, and harbor other genes within its introns. Moreover, because eukaryotic genomes are much more stable over time than those of prokaryotes, eukaryotic genes travel in similar company as they jump from survival machine to survival machine.

An isolated gene pool provides fertile grounds for the rise of complex relationships among genes, resulting in increased epistasis and pleiotropy. The emergence of systems biology from the genomic insights amassed over the past 20 years is in large part devoted to the study of these interactions. Dawkins provides a wonderful analogy through which to understand how selfish genes underpin these networks: imagine teams of rowers that compete in a series of races. To win, rowers must of course interact well with their fellow oarsmen. However, let’s assume that after each race the rowers are randomly reassembled into new teams. If a rower is particularly good and interacts well with most other rowers, she will increase the chances of winning for the teams in which she participates, and thus will win more races in total than does a less able rower. If we were to now organize a new set of races, we might not invite the less successful individuals, and the average speed of boats would go up as a consequence. Likewise, our genes must interact well with the majority of the fellow gene travelers they meet in individual survival machines, and natural selection’s actions on the genes will over time increase the average fitness of individuals.

*The Selfish Gene* continues to be read, yet its most important ideas have not fully pervaded a scientific community that still sees genes as the agents of a species’ coherent genome. One reason may be that the term “selfish gene” itself is such a strong meme (another powerful term introduced by the book) that the complex ideas behind the book’s title are easily forgotten. It is still unnerving how commonly we fall back into putting ourselves — the survival machine — into center stage, when the gene perspective allows a view that is so much clearer. But as long as the concept of survival machines does not feature in textbooks nor at scientific conferences and — most importantly — in the brains of those survival machines, *The Selfish Gene* has not been read enough. Four decades on from its publication, this book is still an amazingly fresh read, and its important insights have not aged. So even if you read it years ago: read it again. Like the genes, which — as Dawkins phrased it — “persist through the generations”, this book also skips down the generations of curious explorers.
